# Supplementary material for: Dedifferentiation of caudate functional organization is linked to reduced D1 dopamine receptor availability and poorer memory function in aging
Source: Imaging Neurosci (Camb). 2025 Jan 31;3:imag_a_00462. doi: 10.1162/imag_a_00462 (PMC12319749; doi:10.1162/imag_a_00462)
Supplement: Supplementary Material [file imag_a_00462-supp.pdf]

Supplementary material

Principal component analysis of memory performance

Table S1. Loadings of individual episodic and working memory tasks on the first principal component that accounted for 54.96% variance in memory performance.

Task	Loading
Word recall	.80
Number-word recall	.64
Object-location recall	.70
Letter updating	.76
Numerical 3-back	.77
Spatial updating	.77

Voxel-wise parcellations of resting-state caudate-cortical connectivity

Table S2. Percentage of caudate voxels allocated to each cortical network in each age group, based on a 7-network parcellation (Yeo et al., 2011).

Network	Young	Middle-aged	Old
Visual network	2.71%	1.70%	2.91%
Somato-motor network	0%	0%	1.30%
Dorsal attention network	0%	0%	1.80%
Ventral attention network	2.10%	3.71%	4.81%
Limbic network	31.76%	34.57%	33.97%
Fronto-parietal network	39.58%	14.13%	10.22%
Default-mode network	23.85%	45.89%	44.99%

Spatial overlap in caudate voxel assignments when using the older adults as the reference group

A similar pattern of findings to that reported in the main manuscript was observed when examining spatial overlap in caudate voxel assignments in the two younger

groups using the older adults as the reference group. Specifically, of the caudate voxels that were allocated to the FPN in the older adults, 85.29% were still allocated to the FPN in the younger adults, whereas 49.02% were allocated to the FPN in the middle-aged adults (44.12% were allocated to the DMN instead in this age group). Of the caudate voxels allocated to the DMN in the older adults, 63.25% were also allocated to the DMN in the middle-aged adults, whereas only 32.74% were allocated to the DMN in the younger adults (44.10% were allocated to the FPN instead in this age group).

Region of interest analyses of resting-state caudate-cortical connectivity using caudate parcels from Choi et al. (2012)

Using the entire caudate parcels previously identified as preferentially coupling with the cortical fronto-parietal (FPN) and default-mode (DMN) networks in Choi et al. (2012) as our caudate regions of interest, we similarly observed a significant interaction between age group (young, middle-aged, older) and cortical target (FPN, DMN) for functional connectivity of the caudate FPN subregion (i.e., the centrolateral caudate) during rest, $F(2,176) = 5.25$, $p = .006$, $\eta_p^2 = .06$ (see Figure S1A). Significantly greater functional connectivity of the caudate FPN subregion with the cortical FPN was observed in the younger adults, $t(58) = 4.47$, $p < .001$, $d = .58$, but not in the middle-aged ($p = .261$) or older adults ($p = .936$), consistent with the seed-based analyses reported in the main manuscript.

In contrast, analysis of resting-state functional connectivity of the caudate DMN subregion indicated a significant main effect of target, $F(1,176) = 37.55$, $p < .001$, $\eta_p^2 = .18$, where greater functional connectivity of the caudate DMN subregion with the cortical DMN than the cortical FPN was observed across participants (see Figure S1B). No evidence for an interaction between age group and target was observed for functional connectivity of the caudate DMN subregion ($p = .473$). However, there was a main effect of age group, $F(2,176) = 3.93$, $p = .021$, $\eta_p^2 = .04$, driven by elevated functional connectivity of the caudate DMN subregion across cortical targets in the middle-aged in comparison to the older adults, $t = 2.80$, $p = .006$, $d = .39$.

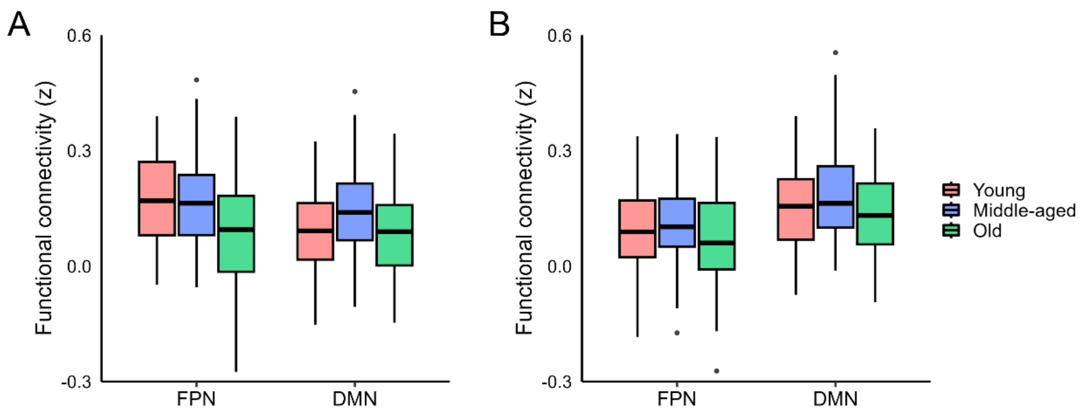

Figure S1. Functional connectivity of the (A) centrolateral and (B) medial wall caudate with the cortical fronto-parietal (FPN) and default-mode (DMN) network in each age group. Caudate subregions were defined based on caudate parcels preferentially coupling with the cortical (A) FPN and (B) DMN in Choi et al. (2012).

Relationship between continuous age and centrolateral caudate functional connectivity

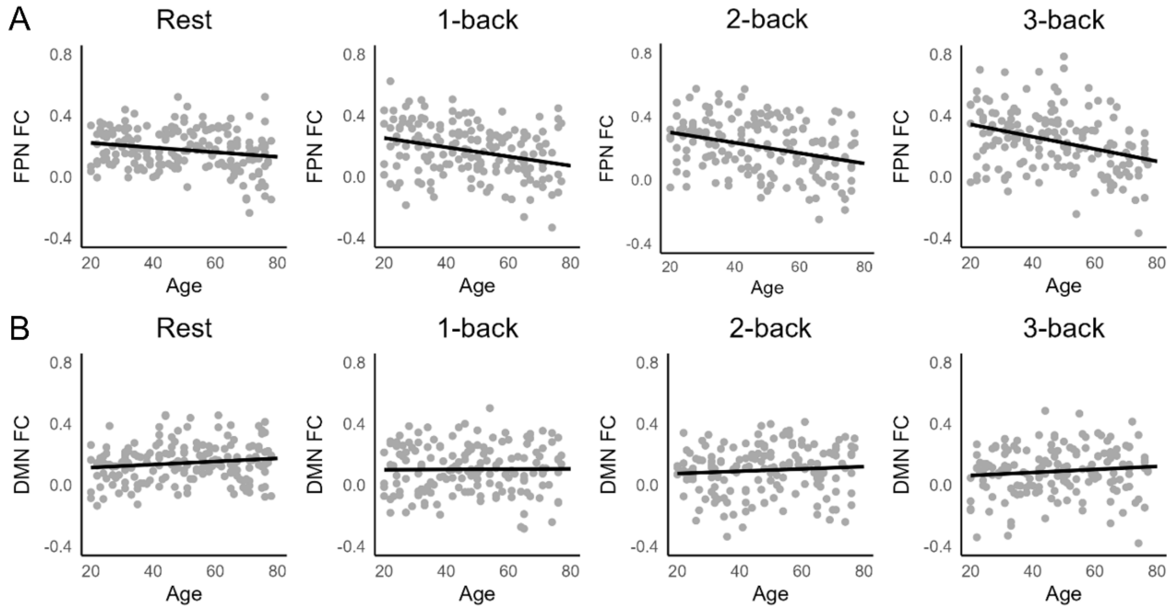

Figure S2. Relationship between continuous age and functional connectivity (FC) of the centrolateral caudate seed with the cortical A) fronto-parietal network (FPN) and B) default-mode network (DMN).

Controlling for educational level in the analyses of functional connectivity and memory associations

Including educational level as an additional covariate in the analyses functional connectivity and memory associations, we similarly observed connectivity differentiation during rest to predict out-of-scanner memory performance across the sample, $\beta = .14$, $SE = .06$, $t = 2.40$, $p = .018$, however, the interaction between age and connectivity became non-significant, $\beta = .08$, $SE = .05$, $t = 1.43$, $p = .154$. Within the age groups, no significant association between connectivity differentiation and memory was still observed in the younger adults, $\beta = .04$, $SE = .13$, $t = 0.27$, $p = .790$, whereas greater differentiation of caudate-cortical connectivity was positively associated with memory performance in the middle-aged, $\beta = .30$, $SE = .13$, $t = 2.39$, $p = .021$, and older adults, $\beta = .34$, $SE = .14$, $t = 2.45$, $p = .018$, even after controlling for education.

For the in-scanner n-back task, a significant 3-way interaction between age, memory load, and connectivity differentiation was similarly observed when including education as an additional covariate in the model, $F(2, 341.86) = 3.53$, $p = .030$. However, the within-group associations between connectivity differentiation and memory during the 3-back condition became non-significant in the middle-aged, $\beta = .21$, $SE = .13$, $t = 1.63$, $p = .110$, and older adults, $\beta = .28$, $SE = .15$, $t = 1.84$, $p = .074$, when additionally controlling for educational level.

Mediation analyses of the effect of age on memory performance via D1DR availability and functional connectivity

We further examined whether the effect of age on memory performance was mediated via D1DR availability and the differentiation of caudate-cortical connectivity, using serial multiple mediation analyses implemented with the R package lavaan (version: 0.6.19). For out-of-scanner memory performance, we considered the potential mediators of caudate D1DR availability, resting-state connectivity differentiation, and caudate D1DR availability via connectivity differentiation. For mean accuracy on the in-scanner n-back task, we examined the potential mediators of prefrontal D1DR availability, mean connectivity differentiation during the n-back task, and prefrontal D1DR availability via connectivity differentiation. 95%

bootstrapped, bias-corrected, confidence intervals were computed based on 5000 samples.

For the analysis of out-of-scanner memory performance, the total indirect effect did not reach significance, β = -.11, 95% CI [-.23, -.01], p = .050, nor did the specific indirect effects of caudate D1DR availability, β = -.05, 95% CI [-.16, .05], p = .333, resting-state connectivity differentiation, β = -.03, 95% CI [-.09, .00], p = .132, or caudate D1DR availability via connectivity differentiation, β = -.03, 95% CI [-.06, .00], p = .070). The direct effect of age was significant, β = -.57, 95% CI [-.72, -.42], p < .001.

Similarly, for the analysis of in-scanner n-back performance, the total indirect effect was not significant, β = -.03, 95% CI [-.14, .07], p = .579, nor were the specific indirect effects of prefrontal D1DR availability, β = .00, 95% CI [-.09, .09], p = .957, connectivity differentiation during the n-back task, β = -.02, 95% CI [-.07, .01], p = .317, or prefrontal D1DR availability via connectivity differentiation, β = -.01, 95% CI [-.03, .00], p = .353. The direct effect of age was significant, β = -.57, 95% CI [-.73, -.41], p < .001.

References

Choi, E. Y., Yeo, B. T. T., & Buckner, R. L. (2012). The organization of the human striatum estimated by intrinsic functional connectivity. *Journal of Neurophysiology*, *108*(8), 2242–2263.

Yeo, B. T. T., Krienen, F. M., Sepulcre, J., Sabuncu, M. R., Lashkari, D., Hollinshead, M., Roffman, J. L., Smoller, J. W., Zöllei, L., Polimeni, J. R., Fischl, B., Liu, H., & Buckner, R. L. (2011). The organization of the human cerebral cortex estimated by intrinsic functional connectivity. *Journal of Neurophysiology*, *106*(3), 1125–1165.
